# Magneto-responsive piezoelectric CoFe_2_O_4_-PVDF system for on-demand electrical modulation of infarcted myocardium

**DOI:** 10.1016/j.mtbio.2026.103500

**Published:** 2026-08-03

**Authors:** Tao Jing, Yaolei Zhang, Zhongtao Li, Xiong Xiong, Hongyu Sun, Yonghong Fan, Chaoming Xie, Feng Lin, Jie Weng, Shuxin Qu

**Affiliations:** a School of Science, Xichang University, Xichang, Sichuan, 615013, PR China; b Institute of Biomedical Engineering, College of Medicine, Southwest Jiaotong University, Chengdu, Sichuan, 610031, PR China; c Clinical Biobank Center, General Hospital of Western Theater Command, Chengdu, Sichuan, 610031, PR China; d School of Life Science and Engineering, Southwest Jiaotong University, Chengdu, Sichuan, 610031, PR China; e Department of Thoracic Surgery, West China Hospital, Chengdu, Sichuan, 610031, PR China

**Keywords:** Magneto-responsive, CoFe_2_O_4_-PVDF, On-demand electrical modulation, Infarcted myocardium, Cardiac therapy

## Abstract

Myocardial infarction (MI) disrupts myocardial electrical conduction and contractile function, causing irreversible cardiac dysfunction. Piezoelectric materials offer great potential for MI repair via in situ electrical generation. However, traditional ultrasound-triggered piezoelectric activation inevitably induces local hyperthermia and secondary myocardial damage. Additionally, the piezoelectric therapeutic mechanisms for cardiac therapy remain poorly understood. Herein, we innovatively fabricated a magneto-responsive, self-powered CoFe_2_O_4_–PVDF composite therapeutic system. This composite achieves on-demand electrical modulation via non-invasive magnetic stimulation: CoFe_2_O_4_ generates magnetostrictive strain under magnetic actuation, which is efficiently converted into functional piezoelectric charges by PVDF, thereby enabling safe, spatiotemporally controllable in situ cardiac electrical stimulation. In vivo results confirmed that the system significantly improved cardiac ejection fraction and reduced myocardial fibrosis. It upregulated key genes governing angiogenesis, calcium signaling, and cardiomyocyte proliferation. In vitro, the composite promoted hypoxic H9c2 cardiomyocyte spreading and suppressed apoptosis under magnetic stimulation. First-principles calculations further verified that CoFe_2_O_4_ optimizes the electronic state density of PVDF and enhances its charge output, revealing the synergistic mechanism. This work establishes an innovative magneto-driven, on-demand electrical therapy platform to overcome the limitations of conventional piezoelectric treatment. It provides novel mechanistic insights and a translational strategy for efficient MI repair via precise in situ regulation of the myocardial electrical microenvironment.

## Highlights

• CoFe_2_O_4_-PVDF on-demand generated the enhanced piezoelectricity at external magnetic field experimental.
• Enhanced piezoelectricity improved the cardiac function and reduced fibrosis area of infracted myocardium *in vivo.*
• CoFe_2_O_4_-PVDF promoted the spreading and reduced the apoptosis of hypoxic H9c2 *in vitro.*
• CoFe_2_O_4_-PVDF upregulated three kinds of genes related to angiogenesis, calcium signal and muscle proliferation.
• CoFe_2_O_4_ enhanced the electronic density and charge output of PVDF at external magnetic field by simulation.

## Graphical abstract

**Figure:**
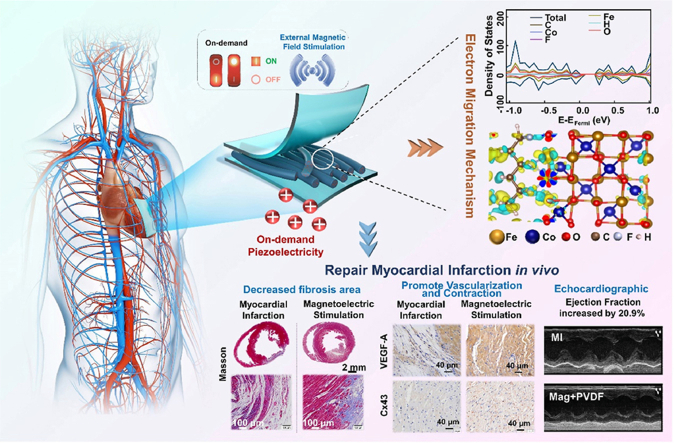

## 1 Introduction

Cardiovascular diseases are a global public health concern [1,2]. Myocardial infarction is a major cause of cardiovascular disease incidence and mortality [3]. It is due to coronary artery occlusion, causing myocardial injury and reduced contractility [4]. Drug therapy has limitations like low myocardial local drug concentration and low bioavailability [5]. Coronary artery bypass grafting can cause ischemia-reperfusion injury [[6], [7], [8]]. In fact, heart contraction begins with sinoatrial node impulses [9]. Gap junction proteins, i.e. Connexin43, in intercalated discs of cardiomyocyte are crucial for transmitting electrical impulses and maintaining electrical coupling [10]. However, after a myocardial infarction, ischemia and hypoxia dephosphorylate connexin channels, reducing the electrical conductivity of myocardium [10,11]. Consequently, enhancing electrical conduction between infarcted and normal myocardium has become a new approach for improving infarcted myocardium recently [12,13]. For instance, the conductive PPy-oxidized xanthan gum prepared can increased the conduction velocity of myocardium [14]. However, the conductive materials only act as a "medium" to improve electrical conduction other than provide extra charges for the infarcted myocardium [15].

Piezoelectric materials can generate electrical charge through mechanical displacements due to non-centrosymmetric crystal structures [16]. Compared to conductive materials, they can provide continuously charges harnessing mechanical forces from bodily movements to provide the extra charges for infarcted myocardium [16]. Among them, polyvinylidene fluoride (PVDF) has been studied in injured myocardium owing to its excellent piezoelectric properties and biocompatibility [17]. Arumugam et al. use PVDF as a cardiac patch for myocardial infarction scar repair [18]. Doustvandi et al. incorporated graphene oxide (GO) into polyvinylidene fluoride (PVDF), yielding an eightfold enhancement in the voltage output of the PVDF-GO composite, demonstrating its potential for application in myocardial repair [19]. Nevertheless, it still lacks an on demand methods to regulate piezoelectric signal although doping GO can prompt PVDF to generate higher polarized charges, because some studies use the physical movement to stimulate the piezoelectric materials. Moreover, ultrasound (US) has be employed to stimulate the piezoelectric materials as a regulator [20]. However, the excessive US intensity might generate extra heat to be detrimental to tissues [20].

Magnetostrictive cobalt ferrite (CoFe_2_O_4_) can expand or contract with changes of external magnetic field [21]. Thus, the strain of doped CoFe_2_O_4_ can stimulate piezoelectric materials to generate piezoelectric charges under an external magnetic field, that is, magnetostrictive CoFe_2_O_4_ and piezoelectric materials can convert the external magnetic field into mechanical energy and further into electrical energy [[22], [23], [24]]. We previously developed CoFe_2_O_4_-PVDF composite. It is anticipated that the strain changes induced by magnetostriction could modulate the piezoelectric performance of piezoelectric materials when exposed to external magnetic field. The proliferation and spread area of doxorubicin-induced oxidative stress injured cardiomyocytes, H9c2 cells, are significantly improved under external magnetic field regulation compared with those without treatment when co-cultured with CoFe_2_O_4_-PVDF, which indicated the convert effect of magnetostrictive-piezoelectric [23]. The possible pathway might be associated the upregulated the "steroid hormone biosynthesis", actuating Na^+^K^+^-ATPase and opening voltage-gated Ca^2+^ channels [23]. Shi et al. propose a novel magnetostrictive-piezoelectric tumor therapy strategy by initiating an intratumoral magneto-driven and piezoelectric-catalyzed reaction using core-shell structured CoFe_2_O_4_-BiFeO_3_ magnetostrictive-piezoelectric nanoparticles under an alternating magnetic field to generate cytotoxic reactive oxygen species (ROS) [24]. These studies show that combining magnetostriction and piezoelectricity enables magnetic-field-controlled piezoelectric modulation, triggering relevant biological effects. In addition, the effect of magnetic fields on various biological systems has been studied [25]. It is shown that different values of magnetic induction and frequency of rotating magnetic field can evoke a different response of cells, e.g., increase in the general metabolic activity, and alter the intracellular Ca^2+^ concentration [26]. Thus, magnetic field is not only to be biosafety but also to modulate the cellular response by optimizing the suitable parameters. Interestingly, it is found that doping CoFe_2_O_4_ can also enhance the piezoelectric effect of piezoelectric materials [23]. Jiang et al. incorporate 5 wt% of CoFe_2_O_4_ into PVDF to enhance piezoelectric performance by approximately 11% [27]. However, the biological influence of CoFe_2_O_4_-PVDF is only verified on cardiomyocytes *in vitro* under external magnetic field [23]. It still lacks the direct evidence to demonstrate that CoFe_2_O_4_-PVDF produce electrical stimulation for repairing injured myocardium *in vivo* and their mechanism is unrevealed.

In this study, the developed on-demand CoFe_2_O_4_-PVDF power-suppliable system was exploring implanted into the model rat for myocardial infarction. Postoperatively, model rats received non-invasive magnetic stimulation to modulate the local electrical microenvironment on demand, as magnetostrictive strain of CoFe_2_O_4_ was converted into piezoelectric charges by PVDF. The therapeutic effects were evaluated via echocardiography and histological assessment. Combined with *in vitro* cell experiments, it was revealed the mechanisms of the on-demand CoFe_2_O_4_-PVDF power-suppliable system in improving infarcted myocardium. Finally, First-principles simulated the calculated electronic state of PVDF irritated by the magnetostriction of CoFe_2_O_4_ under external magnetic field.

## 2 Materials and methods

### 2.1 Preparation of the CoFe_2_O_4_-PVDF power-suppliable system

CoFe_2_O_4_-PVDF was prepared by electrospinning 2 w.t.% of CoFe_2_O_4_ was incorporated into the PVDF precursor solution according to our previous study [23]. The injection rate of the precursor solution, the electrospinning voltage, and the distance between the syringe needle and the collector were 5 mL/h, 18 kV, and 20 cm, respectively. CoFe_2_O_4_ was synthesized using our laboratory method.In previous studies, a series of CoFe_2_O_4_-PVDF composite nanofiber sheets was obtained with various weight contents of CoFe_2_O_4_ at 1, 2, 3 and 4 wt%, which were noted as 1% CoFe_2_O_4_-PVDF, 2% CoFe_2_O_4_-PVDF, 3% CoFe_2_O_4_-PVDF and 4% CoFe_2_O_4_-PVDF, respectively [23].

### *2.2* *In vivo* animal experiments and evaluations

#### 2.2.1 Animal experiments

The experimental rats were divided into 5 groups, which rats were 6 weeks and weighed approximately 200 g. As shown in Table 1 of the Supplementary Materials, except for the sham operation group (Sham), rats in other groups had their left anterior descending coronary artery ligated to establish a myocardial infarction model firstly. Then the other 4 groups were the myocardial infarction group without any treatment (Myocardial Infarction, MI), the daily external magnetic field stimulation group (Magnetic Field, Mag), implanted the CoFe_2_O_4_-PVDF power-suppliable system (PVDF), and implanted the CoFe_2_O_4_-PVDF power-suppliable system combining with external magnetic field stimulation (Mag + PVDF).

**Fig. 1:**
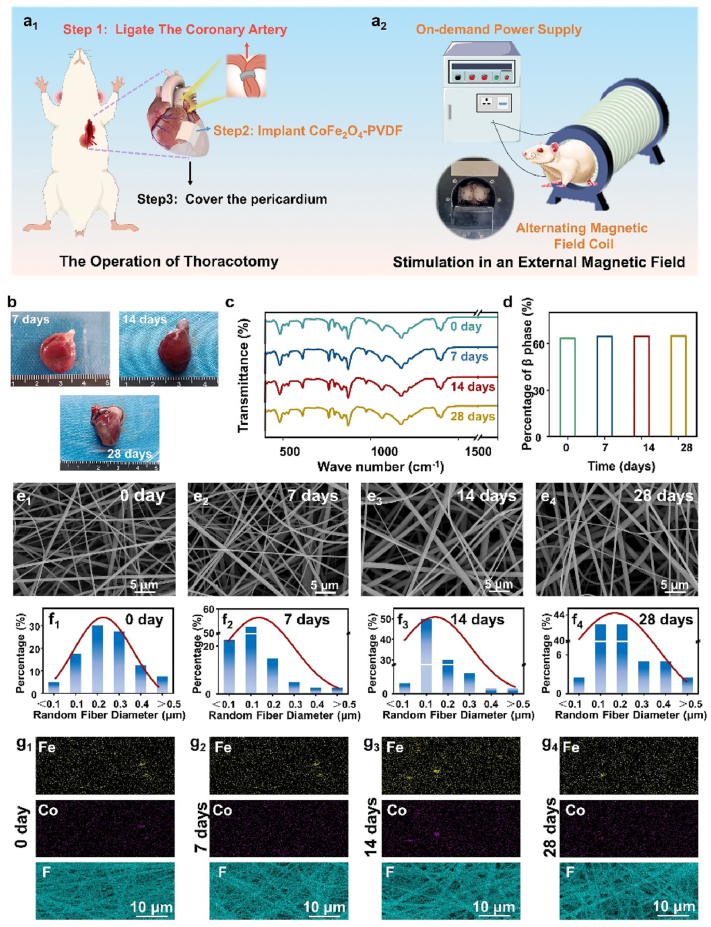
a_1_: The operational steps for implanting the CoFe_2_O_4_-PVDF into the heart of myocardial infarction model rat. a_2_: Schematic illustration of the rat receiving external magnetic field stimulation after the operation. b: The images of stability characterization of CoFe_2_O_4_-PVDF implanted in the rat hearts on 7, 14, and 28 days. Photos of CoFe_2_O_4_-PVDF implanted in rat heart samples; c-d: FT-IR results and the relative content of piezoelectric crystalline β-PVDF; e-f: SEM images and fiber diameter distribution; g: EDS mappings of Fe, Co and F elements in CoFe_2_O_4_-PVDF before and after implantation.

As shown the surgical procedure in Fig. 1a_1_, rats were anesthetized by intraperitoneal injection of pentobarbital sodium at a dose of 50 mg/kg. The rats were fixed in the supine position and connected to a ventilator. The heart was exposed between the third and fourth intercostal spaces of the sternum. The left anterior descending coronary artery was ligated with 0/6 suture 2 mm below the left atrial appendage. Following a gentle disclose of the pericardium, the CoFe_2_O_4_-PVDF was implanted onto the myocardial area with ligated coronary arteries, and the pericardium was subsequently closed over it for fixation. Subsequently, reposition the heart, expel the gas in the thoracic cavity, and suture the incision. In the sham operation group (Sham), the suture passes through the left anterior descending coronary artery but was not ligated. The ethical review of the animal experiment was approved by the Medical Ethics Committee of Southwest Jiaotong University, and the approval number was SWJTU-2403-CDS (082).

After the operation, the experimental group rats were placed in an alternating magnetic field coil to receive magnetic field stimulation for 1 h per day until to 28 days as shown in Fig. 1a_2_. The magnetic field intensity and the alternating frequency were 15 mT and 50 Hz, respectively.

#### 2.2.2 Characterization for CoFe_2_O_4_-PVDF implants *in vivo*

Fourier transform infrared spectrometer (FT-IR, Thermo Scientific Nicolet iS50, USA) with attenuated total reflection (ATR) mode was used to characterize the piezoelectric β-phase of CoFe_2_O_4_-PVDF. The number of scans was 32, the resolution was 4.0 wavenumbers, the acquisition time was 47.3 s, and the laser frequency was 15798.26 cm^−1^. Field emission scanning electron microscope (FESEM, JSM-7800F, Japan) was employed to characterize the morphology of CoFe_2_O_4_-PVDF at 0, 7, 14 and 28 days after implantation onto rat myocardium. Energy dispersive spectrometer (EDS, JSM-7800F, Japan) was employed to analyze the presence of fluorine (F), cobalt (Co), and iron (Fe) in CoFe_2_O_4_-PVDF at 0, 7, 14, and 28 days post-implantation into rat myocardium.

#### 2.2.3 Evaluation of cardiac function indicators

The high-frequency ultrasonic imaging system (Vevo 3100L, FUJIFILM, Japan) was used to evaluate cardiac functions of all experimental rats, such as ejection fraction (EF) and fractional shortening (FS) after operated for 28 days.

#### 2.2.4 Histological staining

The hearts of rats were taken and washed with physiological saline to remove residual blood after they were sacrificed via administration of a lethal dose of pentobarbital sodium. And then fixed with 4% paraformaldehyde solution for 48 h. The fixed myocardium was rinsed in phosphate buffer solution (PBS), gradually dehydrated in ethanol (75%, 85%, 95% and 100%), embedded in paraffin and sectioned with a thickness of 4 μm. The sections were de-waxed twice with xylene at 10 min each time. Then they were hydrated in gradient ethanol (100%, 95%, 85% and 75%) for 3 min in each gradient ethanol, and finally in distilled water for 2 min to eliminate the residual ethanol completely. The sections were stained by Hematoxylin-Eosin (H&E) Masson.

#### 2.2.5 RNA-seq transcriptome sequencing

Total RNA was extracted from myocardial tissue samples of experimental rats. The integrity of the extracted total RNA was detected via agarose gel electrophoresis, and the concentration of RNA was precisely determined. mRNA was isolated from the total RNA, and the captured mRNA was fragmented [28]. The first and second strands of cDNA were synthesized with reverse transcriptase, and the reverse transcription products underwent end repair. Subsequently, a bases were added at the 3′ end, and this fragment was ligated to the sequencing adapter. The ligation products were purified, and the incomplete ligation products were eliminated, followed by PCR amplification using primers complementary to the adapter sequence. Meanwhile, the sequencing library was purified with magnetic beads, and the library concentration was detected by Qubit, while the fragment length of the library was detected by Agilent Fragment Analyzer to guarantee the quality of the library.

#### 2.2.6 Immunohistochemical staining

The method for preparing rat myocardial tissue sections aligned with the methodology detailed in Section 2.2.4 for histological staining. The difference was that immune-histochemical fluorescence vascular endothelial growth factor A (VEGF-A, JH121-MA5-13182, Thermo Fisher Scientific, Germany), vascular endothelial growth factor receptor (VEGF-R, 7422-RBM5-P1ABX, Thermo Fisher Scientific, Germany), and platelet endothelial cell adhesion molecule-1 (CD31, 5175-MSM1-P0, Thermo Fisher Scientific, Germany) were used for immunohistochemical staining.

#### 2.2.7 Immunofluorescence staining

The preparation of myocardial tissue sections was consistent with Section 2.2.4. Then myocardial tissue sections were placed in an acid repair solution and boiled for 2 min. Whereafter, the Conexin43 (Cx43, 71-0700, Thermo Fisher Scientific, Germany), cTNT antibody (cTnT, FXP147, Thermo Fisher Scientific, Germany) and TUNEL (C10617, Thermo Fisher Scientific, Germany) were diluted at 200 times by PBS. Finally, anti-fluorescence quenching agent was dropped for sealing the sections. Photographs were taken with a fluorescence microscope, and the expression changes of the target protein were calculated using Image Pro Plus software. Detailed staining protocols were provided in the Supplementary Materials.

### 2.3 *In vitro* cell experiments and evaluations

#### 2.3.1 Construction and evaluation of the hypoxia model of H9c2 cells *in vitro*

The hypoxic model of H9c2 cells was induced by sodium dithionite treatment (Na_2_S_2_O_4_) [29]. H9c2 cells are an immortalized rat cardiomyoblast cell line, not primary cardiomyocytes; consequently, they retain robust proliferative capacity *in vitro*. In detail, a volume of 1 mM Na_2_S_2_O_4_ was added in DMEM following dissolution and sterile filtration, in which H9c2 cells were induce hypoxic injured after incubated for 4 h to simulate the infarcted myocardial cells. Three experimental groups were established: untreated H9c2 cells (Control), H9c2 cells subjected to hypoxia in the presence of Na_2_S_2_O_4_ (Hypoxia), and hypoxic H9c2 cells co-cultured with CoFe_2_O_4_-PVDF and external magnetic field (Hypoxia + External Magnetic Field). The Hypoxia + External Magnetic Field group was exposed to alternating magnetic field coil (SL34-200, CH-Magnetoelecrticity Technology, China) at 0.5 mT and 50 Hz for 1 h every day.

#### 2.3.2 Cytoskeleton staining

The morphology of the cytoskeleton in H9c2 cells co-cultured with CoFe_2_O_4_-PVDF under an external magnetic field for 3 days was observed. Specifically, the cells were fixed with paraformaldehyde for 15 min, followed by staining with phalloidin for 20 min and DAPI for 5 min. The morphology of H9c2 cells was imaged by Laser Scanning Confocal Microscope (LSCM, A1R+, Nikon, Japan).

Culture endothelial cells (HUVECs) until they reach 70%-80% confluence. Wash the cells twice with phosphate-buffered saline (PBS), then fix them for 15 min at room temperature with PBS containing 4% paraformaldehyde. After fixation, wash the cells three times with PBS. Permeabilize the cells with PBS containing 0.1% Triton X-100 for 10 min at room temperature, followed by three washes with PBS. Incubate the cells in PBS supplemented with 1% bovine serum albumin for 1 h at room temperature. Primary antibody incubation: Place coverslips in blocking buffer and incubate overnight with primary antibodies diluted in blocking buffer (HA1121, ET1705-94 and ET1602-10, HUABio, China). Secondary antibody incubation: Dilute secondary antibodies conjugated with different fluorescent dyes in blocking buffer at a 1:500 ratio and incubate for 1 h at room temperature, protected from light. After three washes with PBS, counterstain cell nuclei with DAPI for 5 min, followed by two additional washes with PBS.

#### 2.3.3 Cell flow cytometry apoptosis evaluation

The density of the H9c2 cell suspension was adjusted to 1 × 10^6^/mL. Firstly, recombinant human Annexin V Alexa Fluor 647 (FXP147 flow cytometry apoptosis kit, SiBoZheng, China) was added dropwise. Then 7-AAD (FXP147 flow cytometry apoptosis kit, SiBoZheng, China) was added dropwise. Finally, PBS buffer was added. Apoptosis was monitored using flow cytometer (TestProfiler, Beckman Coulter, China).

#### 2.3.4 Characterization of magnetic and piezoelectric properties

Field emission scanning electron microscope (FESEM, JSM-7800F, Japan) was employed to characterize the morphology of CoFe_2_O_4_-PVDF. Take CoFe_2_O_4_-PVDF with a mass greater than 10 mg. The magnetic properties of CoFe_2_O_4_-PVDF were characterized by a vibrating sample magnetometer (VSM, HH-15, Nanda, Nanjing, China). The CoFe_2_O_4_-PVDF was cut into 1 cm × 1 cm pieces, and both sides were coated with a 10 nm thick gold layer. Then the charge output signal of CoFe_2_O_4_-PVDF was measured by a linear motor measurement system (NTIAG HS01, LINMOT, USA), and the data were collected through LabView software (National Instruments, USA).

### 2.4 First-principles calculations of the piezoelectricity and electronic states of the CoFe_2_O_4_-PVDF power-suppliable system

The density functional theory (DFT) and the VASP 6.1.0 calculation software [30]. The generalized gradient approximation (GGA) was used to describe the exchange-correlation potential [31]. The projector-augmented wave (PAW) method was used to study the interaction between the ionic core and the valence electrons [32]. The cut-off energy of the plane wave was fixed at 450 eV. The given structural model was relaxed until the Hellmann-Feynman force was less than −0.02 eV/Å and the energy change was less than 10^−6^ eV. The Grimme DFT-D3 method was used to describe the dispersion interactions between atoms in the adsorption model.

### 2.5 Statistical analysis

Triple independent experimental groups were replicated for each quantitative testing. The quantitative data is presented as ‾X ± S. Data were analyzed by one-way analysis of variance (ANOVA), and a p value < 0.05 was regarded as statistically significant. When the p-value is < 0.05, < 0.01 and < 0.001, it is marked with “*”, “**” and “***”, respectively.

## 3 Results

### 3.1 The effect of the CoFe_2_O_4_-PVDF power-suppliable system on improving infarcted myocardium *in vivo*

#### 3.1.1 The stability of CoFe_2_O_4_-PVDF *in vivo*

As in Fig. 1a and Fig. S1 show the schematic and real surgical procedures to establish the myocardial infarction model rat, implanting CoFe_2_O_4_-PVDF into its heart, and stimulating the rat with the CoFe_2_O_4_-PVDF power-suppliable system under an external magnetic field postoperatively, respectively. In detail, it included thoracotomy, and ligation of left anterior descending coronary artery until the left ventricle anterior wall myocardial tissue turns pale or cyanotic, which were regarded as the feature of myocardial infarction. Thus, it was established the myocardial infarction model rat. Then CoFe_2_O_4_-PVDF were implanted in infarcted myocardium and subsequently covered with the myocardial membrane. Finally, the wound of rat was sutured layer by layer. Rats after the surgery received external magnetic field stimulation therapy for 30 min every day. Fig. 1b presents the physical photographs of the CoFe_2_O_4_-PVDF material harvested on the 7, 14, and 28 days after its implantation into the hearts of myocardial infarction model rat. The FT-IR results of implanted CoFe_2_O_4_-PVDF materials are shown in Fig. 1c. The characteristic absorption peak of the piezoelectric crystal form β-PVDF is at a wavenumber of 840 cm^−1^ [33]. The intensity of the absorption peak at this wavenumber did not decrease even until to 28 days after implanted. Furthermore, the quantitative relative contents of the piezoelectric β-PVDF crystal remained nearly identical, approximately 64%, after implanted for 7, 14, and 28 days compared to that of without implantation as shown in Fig. 1d. Fig. 1e presents the SEM results of the CoFe_2_O_4_-PVDF power-suppliable system implanted in of myocardial infarction model rat for 0, 7, 14, and 28 days, respectively. The CoFe_2_O_4_-PVDF materials still displayed a fibrous structure and retained a distinct fibrous morphology until to 28 days after being implanted in the rat myocardium. Furthermore, the fiber diameter distributions of CoFe_2_O_4_-PVDF materials were measured after implanted for 0, 7, 14, and 28 days, as depicted in Fig. 1f. The fiber diameter distributions were nearly identical with a diameter range of 100 ∼ 200 nm after implanted for different time points. Fig. 1g presents the EDS results of the CoFe_2_O_4_-PVDF materials at various *in vivo* implantation times. After 28 days of implantation into the heart of myocardial infarction model rat, the Co and Fe elements remained detectable. For instance, EDS semi-quantitative results showed that the mass percentages of Co element were 0.32 w.t.% and 0.26 w.t.% before and after implantation into the rat myocardium as an example, respectively. The *in vivo* and *in vitro* biocompatibility evaluation of CoFe_2_O_4_-PVDF is shown in Figs. S3, S4, and S5 of the Supplementary Materials. First, Fig. S3 shows the Live/Dead staining fluorescence images of oxidative stress injured H9c2 cells co-cultured with CoFe_2_O_4_-PVDF composite nanofiber sheets, PVDF and the blank control for 3 d. There were many living cells and few dead cells, which were stained with green and red fluorescence either with or without magnetic field cue, respectively. Furthermore, as shown in Fig. S4, after 4 h of co-incubation with fresh anticoagulated rat blood, the positive control displayed a uniformly red supernatant, indicative of complete hemolysis and hemoglobin release. In contrast, both the CoFe_2_O_4_-PVDF extract group and the negative control yielded colorless, transparent supernatants with intact erythrocyte pellets at the tube bottom, confirming negligible hemolysis and thus excellent hemocompatibility. Finally, histopathological examination of lung, liver, spleen, and kidney tissues harvested 28 days post-implantation revealed no inflammatory infiltration, fibrotic deposition, or disruption of native tissue architecture (Fig. S5), collectively supporting the systemic biocompatibility of CoFe_2_O_4_-PVDF. Furthermore, the *in vitro* release of Co and Fe was carried out as degradation testing as shown in Fig. S8. It indicated that CoFe_2_O_4_-PVDF were hardly degradable, which was benefit for provide the good biocompatibility of CoFe_2_O_4_-PVDF.

#### 3.1.2 The effect of CoFe_2_O_4_-PVDF on improving infarcted myocardium *in vivo*

**Fig. 2:**
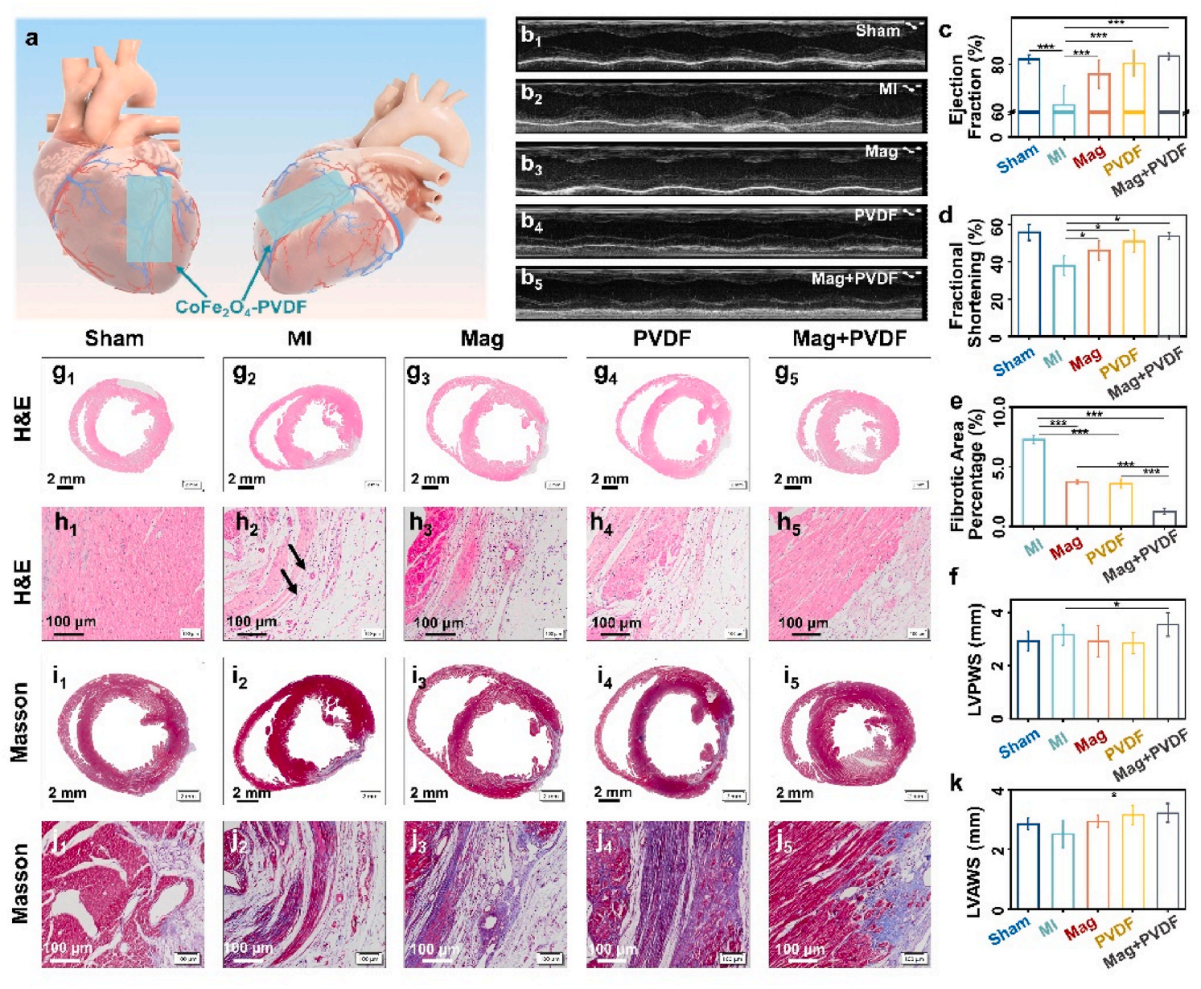
Cardiac function indexes and histological section results after implanted CoFe_2_O_4_-PVDF power-suppliable system into the heart of myocardial infarction model rats. a: Schematic diagram of the implanted position of CoFe_2_O_4_-PVDF power-suppliable system *in vivo*; b: Echocardiographic images; c: the quantitative data of Ejection fraction; d: the quantitative data of Left ventricular fractional shortening; e: Quantitative statistical results of myocardial fibrosis area; f: the quantitative data of Left ventricular posterior wall end-systolic diameter (LVPWS); g-h: Hematoxylin-Eosin staining (H&E) staining results; i-j: Masson staining results; k: the quantitative data of Left ventricular anterior wall end-systolic diameter (LVAWS). “*” indicates p < 0.05, “**” indicates p < 0.01 and “***” indicates p < 0.001, N = 6.

Fig. 2a shows the position where the CoFe_2_O_4_-PVDF power-suppliable system is implanted into the heart of myocardial infarction model rat. The echocardiography results of rats are presented in Fig. 2b for all groups. Ejection fraction is the percentage of blood ejected from the left ventricle during each contraction relative to its total volume at the end of diastole. Fractional shortening is a linear measurement of left ventricular contractility, representing the percentage change in left ventricular internal diameter from diastole to systole. Fig. 2c–f presents the quantitative cardiac function index of ejection fraction and left ventricular fractional shortening, myocardial fibrosis area and left ventricular posterior wall end-systolic diameter (LVPWS) for rats in each group after operated for 28 days derived from those as in Fig. 2b, respectively. The quantitative data of the fibrotic areas are presented in Fig. 2e, indicating that the CoFe_2_O_4_-PVDF power-supplying system in conjunction with external magnetic field can improve the repairing of the infarcted myocardium. The ejection fraction, left ventricular fractional shortening, LVAWS and LVPWS of rats were significantly higher in the Mag + PVDF group than those of rats in the myocardial infarction group (MI) without the stimulation of the CoFe_2_O_4_-PVDF power-suppliable system combined with external magnetic field. These results demonstrated that the CoFe_2_O_4_-PVDF power-suppliable system combination with the external magnetic field in can significantly improve the cardiac function of the myocardial infarction model rats. Notably, sustained external magnetic stimulation of CoFe_2_O_4_-PVDF for 28 days, relative to the day-14 d, elicited statistically significant improvements in ejection fraction, left ventricular fractional shortening, and cardiac output, indicating progressive recovery of systolic function.

Fig. 2g and h shows the results of H&E staining results of rat myocardium and the locally magnified images. As shown in Fig. 2g_1_ and 2h_1_, the normal rat myocardium was stained as a uniform dark red, indicating the intact tissue structure as homogeneous eosinophilic staining. The sarcoplasm of cardiomyocytes formed an interconnected syncytial network [34,35]. Although the morphology of the cross-sectional myofilament bundles was irregular, there was no separation among adjacent myofilament bundles, and they still were continuous with each other. The overall myocardium was plump and compact.

In contrast, the other groups all shows heterogeneous red staining with some pale or lighter red zones, suggesting the presence of fibrotic areas as shown in Fig. 2g_2_-g_5_ and 2h_2_-h_5_, which confirmed that ligating the left anterior descending coronary artery successfully established a myocardial infarction model in rats. Myocardial cells showed signs of shrinkage, with the leaking of nuclear substances from the injured cells and aggregating into dense masses. In addition, disintegration was observed at the junctions between apoptotic myocardial cells and adjacent cells, leading to the formation of apoptotic bodies (position at arrow). Nevertheless, the Mag + PVDF group displayed more densely arranged myocardial tissue and a marked reduction a pale red zones as fibrotic areas compared with that of the MI group after implanted the CoFe_2_O_4_-PVDF power-supply system combined with daily external magnetic field treatment for 28 days as presented in Fig. 2g_5_ and 2h_5_.

The results of Masson staining are presented in Fig. 2i and j. In normal rats (Sham), the myocardium mainly was dense and appeared as red, which exhibited the characteristics of a normal myocardial cross-section as depicted in Fig. 2i_1_ and 2j_1_. In the Masson staining sections of the other groups, there were large blue areas due to the new-formed of collagen in the injured myocardium as shown in Fig. 2j_2_-j_5_. Similarly, there were more normal red staining area in Mag + PVDF group compared with that of the other groups after implanted the CoFe_2_O_4_-PVDF power-supply system and treated external magnetic field every day for 28 days as depicted in Fig. 2j_5_. Simultaneously, the partial blue fibrotic area was remarkably reverted to the typical red coloration, indicating the repairing of injured myocardium.

### 3.2 The mechanism of CoFe_2_O_4_-PVDF to improve infarcted myocardium

**Fig. 3:**
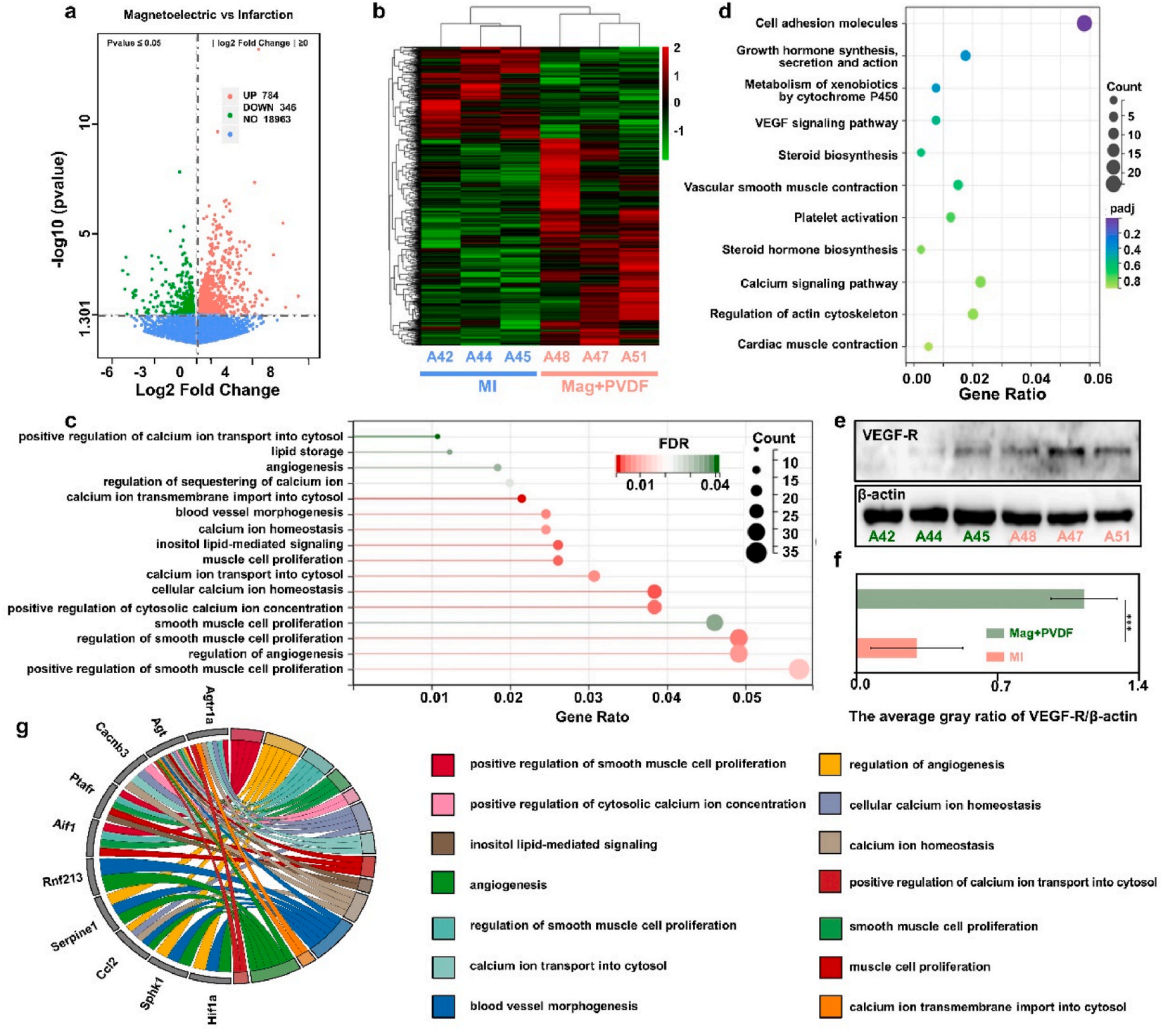
Transcriptome and Western blotting results of infarcted myocardium in model rats treated by the CoFe_2_O_4_-PVDF power-suppliable system and external magnetic field. a: A volcano plot of differentially expressed genes; b: Clustering heatmap of differentially expressed genes; c: Upregulated pathways of GO biological process functional enrichment; d: Enrichment results of upregulated KEGG signaling pathways. e-f: Western blotting bands and gray ratio results of VEGF-R and β-action proteins. MI represents the control group of myocardial infarction, and Mag + PVDF represents the group of myocardial infarction improved by the stimulation of the CoFe_2_O_4_-PVDF power-suppliable system in combination with the external magnetic field. g: Graph of GO enrichment statistical results for angiogenesis-related genes. “*” indicates p < 0.05, “**” indicates p < 0.01 and “***” indicates p < 0.001, N = 3.

Fig. 3a presents a volcano plot of differentially expressed genes in infarcted myocardium from model rats treated with the CoFe_2_O_4_-PVDF power-suppliable system and external magnetic field. The results indicated that there were 784 upregulated genes and downregulated 346 genes in myocardial infarction rats. Fig. 3b presents a clustering heatmap of myocardial infarction rats. The analysis revealed pronounced intra-group clustering, demonstrating that the transcriptional alterations were highly concordant among samples within the same group. Fig. 3c shows the results of the enriched upregulated GO biological process relative signaling pathways in myocardial infarction rats treated with the CoFe_2_O_4_-PVDF power-suppliable system and external magnetic field stimulation. In Mag + PVDF group, the genes associated with the biological processes of “blood vessel morphogenesis”, “angiogenesis” and “regulation of angiogenesis” were significantly upregulated compared with those of MI group. In addition, it is up-regulated for “Steroid biosynthesis”, “calcium signaling” and “muscle” related genes. Fig. 3d presents the results of the upregulated KEGG signaling pathways in Mag + PVDF group. The differentially expressed genes in Mag + PVDF group were significantly enriched in the “VEGF signaling pathway”, “calcium signaling pathway” and “muscle cell proliferation pathway” compared with those of MI group. Fig. 3e presents the Western blotting results of the VEGF-R receptor protein in rats with myocardium treated by the CoFe_2_O_4_-PVDF power-supplying system and external magnetic field stimulation. The VEGF-R band intensities were notably enhanced in the Mag + PVDF group compared to those in the MI group. Subsequently, a quantitative analysis was performed by comparing the gray scale ratio with the internal reference β-actin. As depicted in Fig. 3f, the gray value ratio of the VEGF-R protein band in Mag + PVDF group was 1.31 by comparing with to that the internal reference protein β-actin, whereas that in the control group (MI) was 0.30. Fig. 3g displays the GO enrichment statistics for angiogenesis-related genes. Genes associated with angiogenesis or vascular contraction, including Agtr1a, Agt and Cacnb3, were subjected to GO pathway enrichment analysis. Pathways such as regulation of angiogenesis and blood vessel morphogenesis were highly co-enriched by these genes, implying that ABC and other genes may cooperatively regulate vascular development or remodeling.

**Fig. 4:**
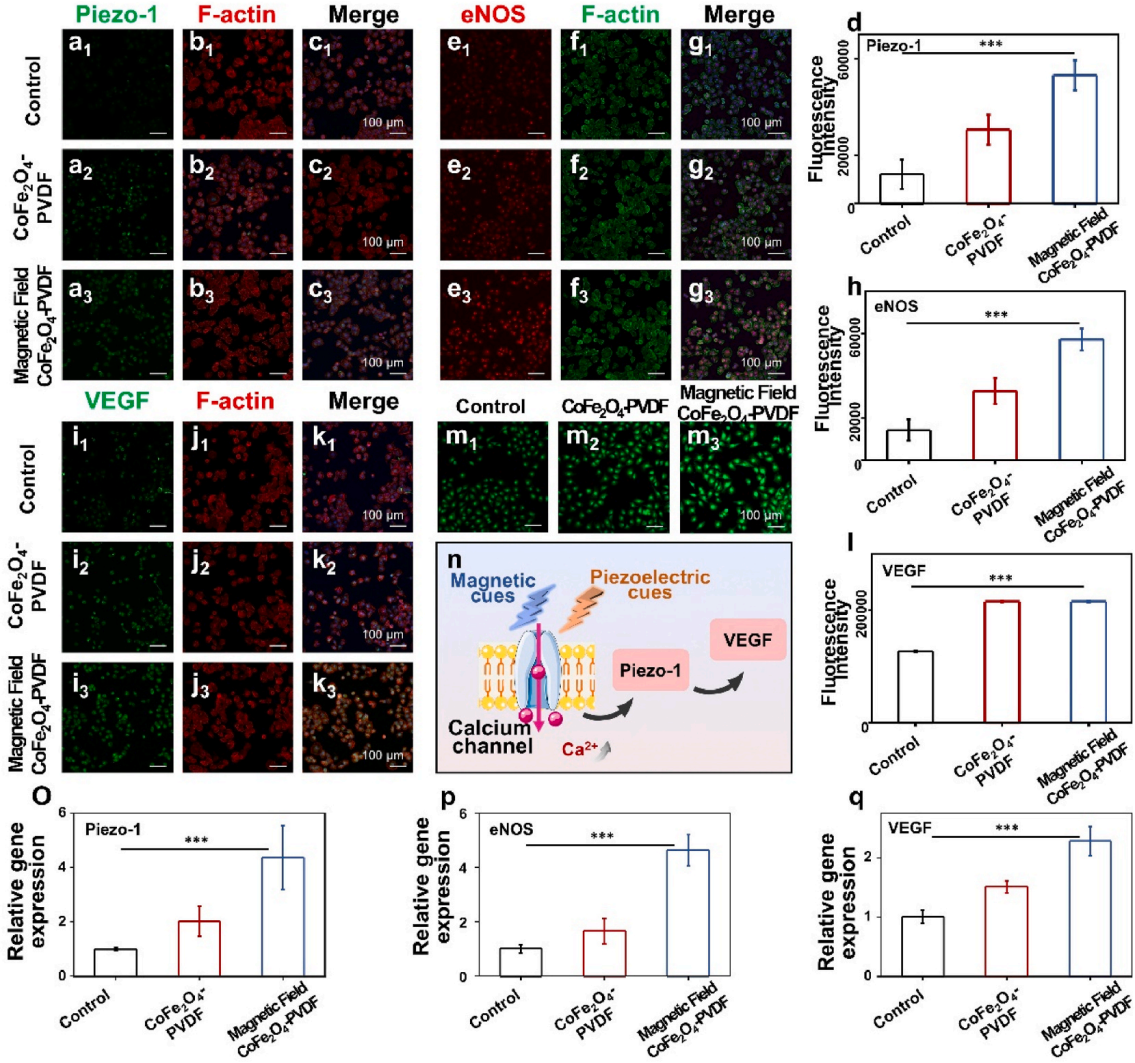
Immunofluorescence characterization of endothelial cell responses to magnetoelectric stimulation. 4a-4c: Piezo1 immunofluorescence staining; 4d: Quantification of Piezo1 fluorescence intensity. 4e-4g: eNOS immunofluorescence staining; 4h: Quantification of eNOS fluorescence intensity. 4i-4k: VEGF immunofluorescence staining; 4l: Quantification of VEGF fluorescence intensity. 4m: Intracellular Ca^2+^ imaging using Fluo-4 AM staining following magnetoelectric stimulation. 4n: Proposed mechanistic model integrating Piezo1-mediated mechanotransduction, calcium signaling, and downstream angiogenic activation. 4o-4q: Quantitative PCR analysis of Piezo1, eNOS, and VEGF mRNA expression levels in HUVECs. “*” indicates p < 0.05, “**” indicates p < 0.01 and “***” indicates p < 0.001, N = 3.

Fig. 4a–c displays representative immunofluorescence images of Piezo1 (a mechanosensitive cation channel) in HUVECs, specifically from the CoFe_2_O_4_-PVDF group under external magnetic stimulation. Fig. 4d quantifies the fluorescence intensity of Piezo1 staining, demonstrating a statistically significant upregulation relative to both blank and PVDF-only control groups. Fig. 4e–g shows representative immunofluorescence images of endothelial nitric oxide synthase (eNOS), and Fig. 4h presents corresponding fluorescence intensity quantification, revealing robust and significant eNOS inductionunder magnetoelectric stimulation. Similarly, Fig. 4i–k depict VEGF immunofluorescence staining, with quantitative analysis in Fig. 4l confirming marked VEGF upregulation. Finally, Fig. 4m shows intracellular Ca^2+^ dynamics visualized by Fluo-4 AM staining; subpanel 4m_3_ (CoFe_2_O_4_-PVDF + magnetic field) exhibits markedly intensified fluorescence compared with the control (4m_1_), consistent with enhanced calcium influx. All data are representative of at least three independent experiments. Fig. 4o–q presents quantitative PCR analysis of Piezo1, eNOS, and VEGF mRNA expression levels in HUVECs. Exposure to the external magnetic field in combination with CoFe_2_O_4_-PVDF significantly upregulated the transcription of all three genes relative to the untreated control group.

**Fig. 5:**
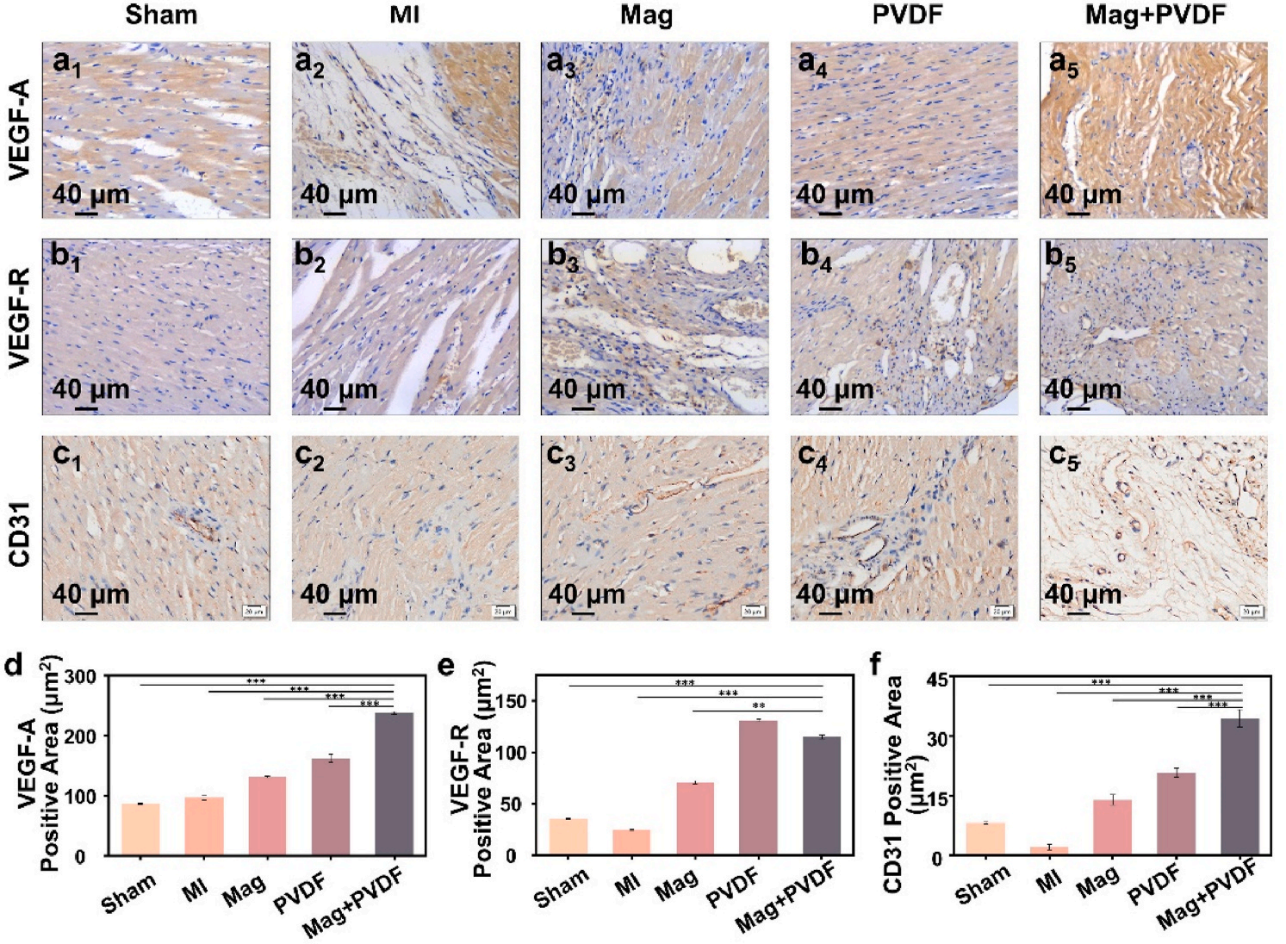
Immunohistochemical staining results of cardiac tissues in myocardial infarction rats treated with the CoFe_2_O_4_-PVDF power-suppliable system and external magnetic field stimulation. a-c: Staining of vascular endothelial growth factor A (VEGF-A), vascular endothelial growth factor receptor (VEGF-R), and Platelet endothelial cell adhesion molecule-1 (CD31) and d-f: Quantitative statistical results of positive areas. “*” indicates p < 0.05, “**” indicates p < 0.01 and “***” indicates p < 0.001, N = 6.

Fig. 5a–5c display the immunohistochemical staining images of vascular endothelial growth factor A (VEGF-A), vascular endothelial growth factor receptor (VEGF-R), and Platelet endothelial cell adhesion molecule-1 (CD31) in the myocardium sections of rats respectively. The brown areas in Fig. 5a–5c represent the positive regions of VEGF-A, and VEGF-R, and CD31 in rat myocardium, respectively. The blue color represents the cell nuclei stained with Hematoxylin to distinguish a clearer cellular contours.

Fig. 5a_2_, 5b_2_, and 5c_2_ show immunohistochemical staining images of infarcted myocardium of model rats, in which the positive areas of VEGF-A, VEGF-R, and CD31 were the smallest all groups. After treated with the CoFe_2_O_4_-PVDF power-supplying system and external magnetic field for 28 days, the positive area of VEGF-A, VEGF-R and CD31 obviously increased in Mag + PVDF groups as depicted in Fig. 5a_5_, 5b_5_ and 5c_5_. Furthermore, the quantitative positive areas of VEGF-A, VEGF-R, and CD31 were showed in Fig. 5d–5f, respectively. The expressions of these three proteins in Mag + PVDF group were the highest in all groups.

**Fig. 6:**
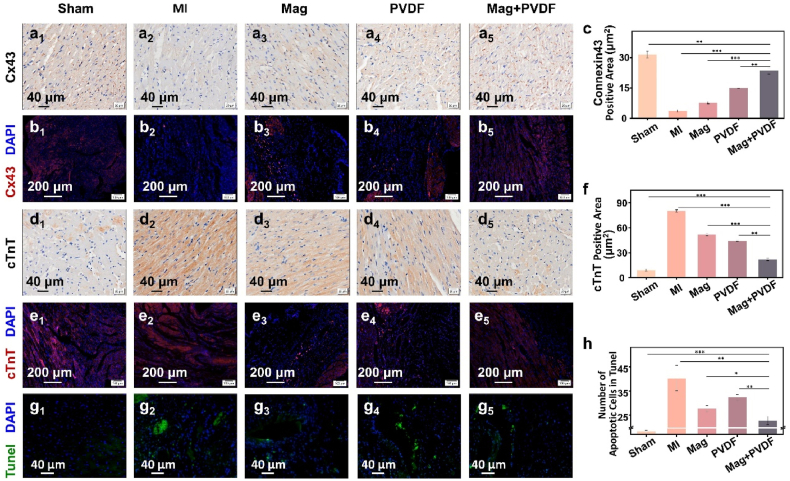
Immunohistochemistry and TUNEL fluorescence staining images of cardiac tissues in myocardial infarction rats treated with the CoFe_2_O_4_-PVDF power-suppliable system and external magnetic field stimulation. a-c: Staining of gap junction protein 43 (Connexin43, Cx43) and quantitative results of positive area; d-f: Staining of cardiac troponin T (cTnT) and quantitative results of positive area; g-h: Apoptosis TUNEL fluorescence staining images and quantitative results of the number of positive cells. “*” indicates p < 0.05, “**” indicates p < 0.01 and “***” indicates p < 0.001, N = 6.

Fig. 6a and b display the immunohistochemical and immunofluorescence staining results of gap junction protein 43 (Connexin43, Cx43) in rat myocardium, respectively. The brown color in Fig. 6a and the red color in Fig. 6b represent the positive regions of Connexin43 in rat myocardium, respectively. There were the largest positive areas of Connexin43 in sham group, followed by the Mag + PVDF group. The brown positive areas in these two groups were significantly larger than those in the other groups. Fig. 6c presents the quantitative statistical results of Connexin43 in the myocardium of all groups, which the positive area in Mag + PVDF group was significantly larger than that in MI, Mag and PVDF groups. Fig. 6d and e displays the immunohistochemistry and immunofluorescence staining results of cTnT in rat myocardium, in which the brown color in Fig. 6d and the red color in Fig. 6e represent the positive regions of cTnT in rat myocardium, respectively. As shown in Fig. 6d, the area of cTnT positive signals in the Mag + PVDF group was obviously decreased. The quantitative statistics indicated that the positive area of cTnT positive markers in rats of the Mag + PVDF group was significantly smaller than that in MI, Mag and PVDF groups as shown in Fig. 6f. In Fig. 6g, the green color corresponds to apoptotic positive cells, while the blue color represents the nuclei of cells. The quantitative statistical results indicated that the number of positive cells in Mag + PVDF group was significantly lower than that of MI, Mag and PVDF groups, whereas the number of positive cells of MI group was the highest in all groups as presented in Fig. 6h.

### 3.3 The CoFe_2_O_4_-PVDF power-suppliable system improve infarcted H9c2 cells *in vitro*

**Fig. 7:**
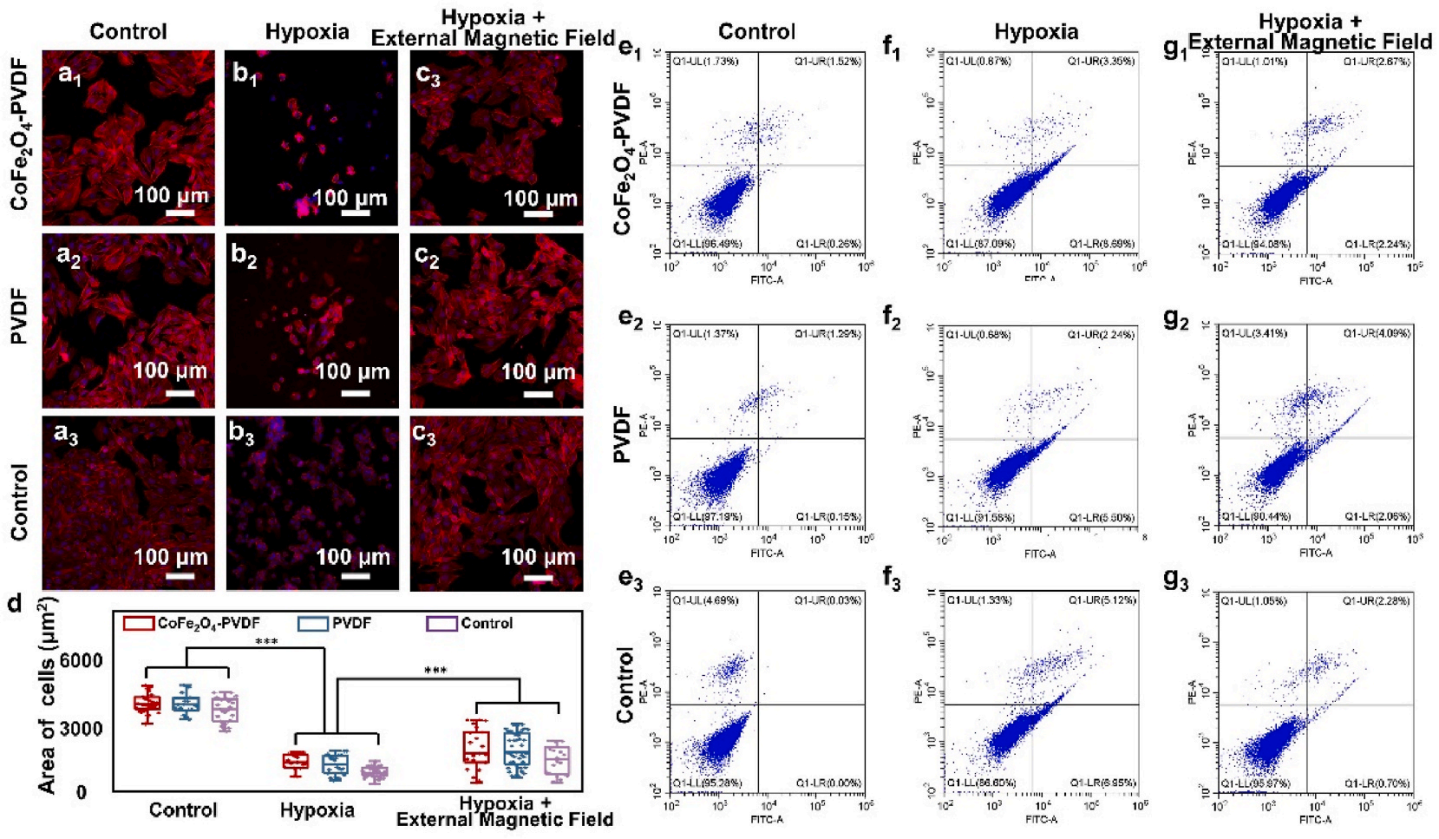
Results of CoFe_2_O_4_-PVDF materials in combination with external magnetic field to improve hypoxic H9c2 cells. a-c: The cytoskeleton staining images of various H9c2 cells, the blank normal H9c2 cells group (Control), hypoxia group (Hypoxia), and magnetoelectric stimulation group after hypoxia (Hypoxia + External Magnetic Field); d: Quantitative measurement results of the spreading area of cells in each group; e-g: Flow cytometry apoptosis results. “*” indicates p < 0.05, “**” indicates p < 0.01 and “***” indicates p < 0.001, N = 4.

Fig. 7 shows the results of hypoxic H9c2 cells co-cultured with the CoFe_2_O_4_-PVDF power-suppliable system with or without external magnetic field stimulation *in vitro*. Fig. 7a–7c show the cytoskeleton staining images of the control normal and hypoxic H9c2 cells with or without an external magnetic field stimulation. The application of an external magnetic field stimulation led to obvious increase in the cytoskeletal staining area of hypoxic H9c2 cells co-culture either with CoFe_2_O_4_-PVDF or with PVDF, although all were significantly lower than that of the normal control group. As depicted in Fig. 7b, the size of hypoxic H9c2 cells without external magnetic field stimulation appeared smaller compared with that of the control and the hypoxic with external magnetic field stimulation, which might be due to the cellular shrinkage. Particularly, the normal morphology of hypoxic H9c2 cells could be sustained in the hypoxia group co-cultured with the PVDF or CoFe_2_O_4_-PVDF power-suppliable system in conjunction with external magnetic field stimulation as presented in Fig. 7c. As shown in Fig. 7d, the quantitative data of the cell spreading areas demonstrated that the control group exhibited the largest area, followed by the hypoxia group with external magnetic field stimulation, while the hypoxia group without stimulation showed the smallest area. Quantitative analysis revealed a significant reduction in cell spreading area under hypoxia, which was partially rescued by external magnetic field stimulation.

Furthermore, the apoptosis of hypoxic H9c2 cells was assessed by flow cytometry following treatment with the CoFe_2_O_4_-PVDF power-supplying system under an external magnetic field, as shown in Fig. 7e–g. In the resulting apoptosis profiles, the abscissa indicates the fluorescence intensity of Annexin V-FITC, with higher values corresponding to a greater extent of early apoptosis. The ordinate represents the fluorescence intensity of propidium iodide (PI), where increased signal reflects more advanced apoptotic stages or cell death. In Fig. 7f_1_, the number of apoptotic cells in the hypoxia group (Hypoxia) was notably higher than that in the normal H9c2 cells group (Control), which indicated the successful construction of a hypoxic H9c2 cell model. In Fig. 7g_1_, the number of apoptotic cells was significantly lower than that in the hypoxia group. For quantitative analysis, the apoptosis of hypoxic H9c2 cells in each group was determined by calculating the combined sum of late apoptotic/dead cells (upper right quadrant) and early apoptotic cells (lower right quadrant) in Fig. 7e–g. Among them, the apoptosis rates were 1.78%, 10.04%, and 4.91%, for the H9c2 normal cells group, hypoxia group, and hypoxia with the CoFe_2_O_4_-PVDF power-supplying system combined with external magnetic field group, respectively. Specifically, co-cultured with the CoFe_2_O_4_-PVDF power-supplying system combined with external magnetic field significantly decreased the apoptosis rate of hypoxia H9c2 cells by 5.13%. These results indicated that the CoFe_2_O_4_-PVDF power-supplying system combined with external magnetic field can reduce the apoptosis of hypoxia H9c2 cells, which might be potential for improving infarcted myocardium *in vivo*.

**Fig. 8:**
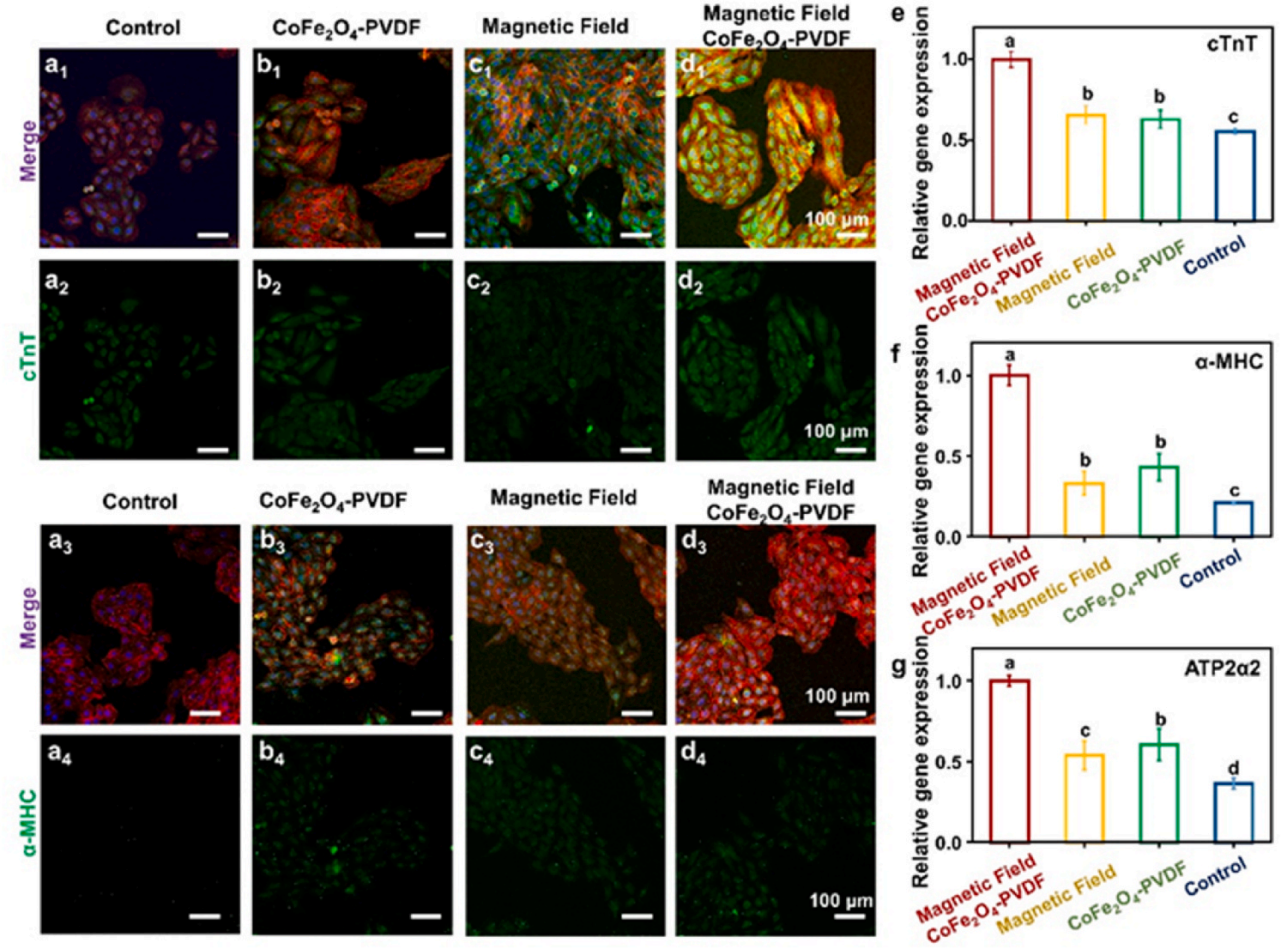
Verification of the synergy of the CoFe_2_O_4_-PVDF power-suppliable system and the external magnetic field in promoting the contractile function of H9c2 cells. a, b, c and d represent the immunofluorescence staining of troponin cTnT and myosin α-MHC in the experimental group (Magnetic Field + CoFe_2_O_4_-PVDF), the external magnetic field group (Magnetic Field), the piezoelectric group (CoFe_2_O_4_-PVDF), and the blank group (Control), respectively. e, f and g represent the PCR results of cTnT, α-MHC and ATP2α2 genes of rat myocardial H9c2 cells, respectively.

Fig. 8a–8d display the immunofluorescence staining results of troponin cTnT and myosin α-MHC in injured H9c2 cells. These results indicate that the situation is improved by the CoFe_2_O_4_-PVDF power-suppliable system in combination with external magnetic field. The method for establishing injured cells is detailed in the Supplementary Materials. In Fig. 8a1 and 8a3, the cytoskeleton of H9c2 cells is represented by the red color, while the cell nucleus is represented by the blue color. In Fig. 8a2 and 8a4, the protein expressions of cTnT and α-MHC are represented by the green fluorescence, respectively. The cells in the CoFe_2_O_4_-PVDF power-suppliable system combined with external magnetic field group exhibit the highest immunofluorescence intensities of cTnT and α-MHC. Fig. 8e, f, and 8g respectively display the gene expression results of cTnT, α-MHC, and ATP2α2 in H9c2 cells *in vitro* under the combined effect of the CoFe_2_O_4_-PVDF power-supplying system and external magnetic field. The ATP2α2 gene encodes the sarco/endoplasmic reticulum Ca^2+^-ATPase 2 (SERCα2), a critical calcium-handling protein in cardiac myocytes. In all three scenarios, the expression was significantly upregulated in the group with the CoFe_2_O_4_-PVDF power-supplying system combined with external magnetic field. Moreover, there was a more than twofold significant difference compared to the Control group of injured H9c2 cells.

### 3.4 An on-demand charge generation in the CoFe_2_O_4_-PVDF power-suppliable system in response to an external magnetic field

**Fig. 9:**
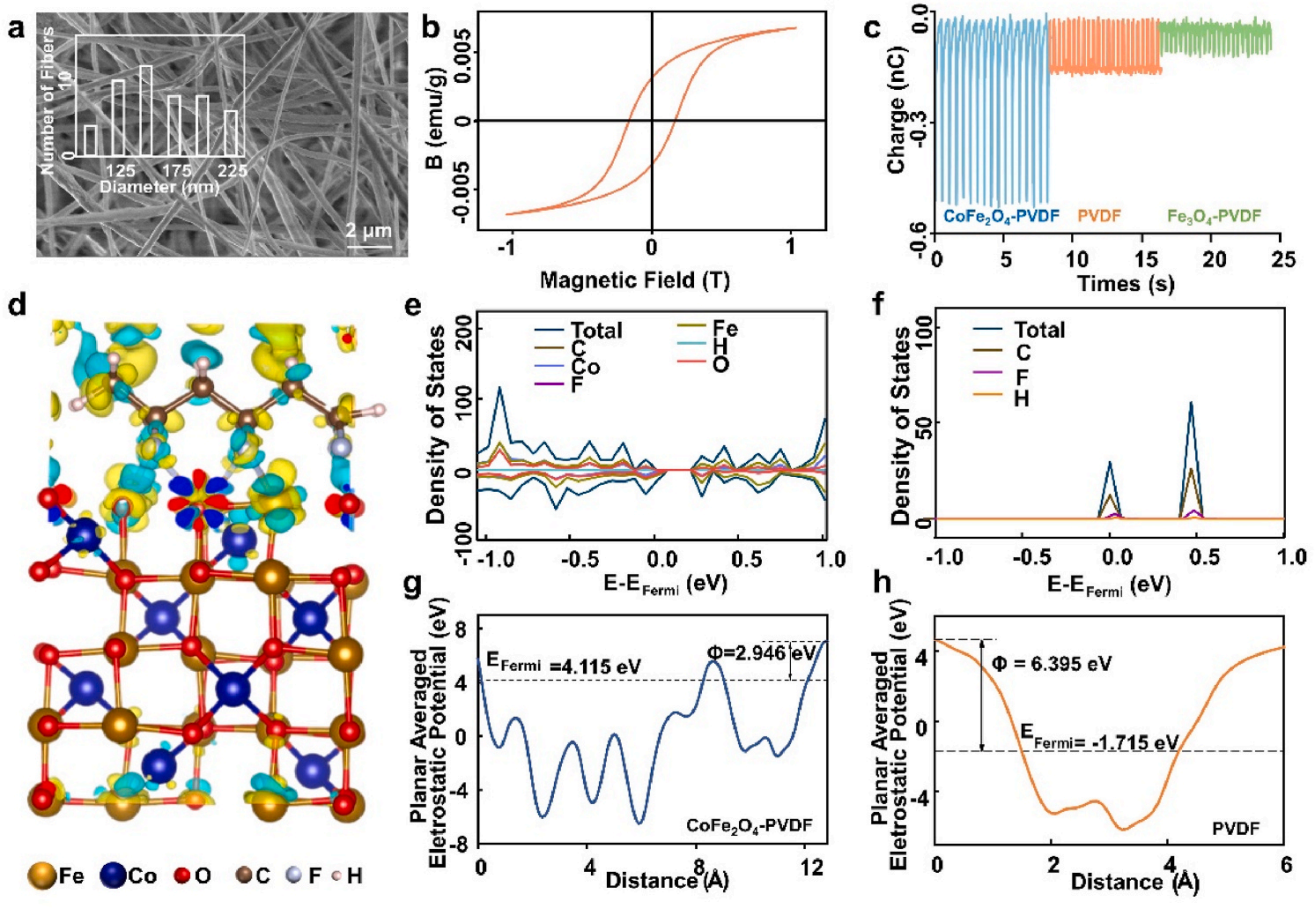
The characterization and First-principles simulative calculating results of CoFe_2_O_4_-PVDF. a: SEM results; b: VSM results and c: piezoelectric charge output results of CoFe_2_O_4_-PVDF. d: The differential charge density diagram of CoFe_2_O_4_-PVDF; e-f: the density of states of CoFe_2_O_4_-PVDF and PVDF, respectively; g-h: the work functions of CoFe_2_O_4_-PVDF and PVDF, respectively.

Fig. 9a–9c present the SEM image, magnetic and piezoelectric properties of the CoFe_2_O_4_-PVDF materials. The CoFe_2_O_4_-PVDF materials displayed a fibrous structure with an average diameter of 150 nm as shown in Fig. 9a and the inset. The magnetic hysteresis loop of the CoFe_2_O_4_-PVDF materials is shown in Fig. 9b, where the applied magnetic field (abscissa) is plotted against the measured magnetization (ordinate). The CoFe_2_O_4_-PVDF materials showed a saturation magnetization of 0.0046 emu/g, confirming its magnetic character. Fig. 9c depicts the piezoelectric properties of the CoFe_2_O_4_-PVDF, showing its charge output in response to applied pressure. For comparison, pure PVDF and non-magnetostrictive Fe_3_O_4_-PVDF without magnetostrictive properties were tested as controls. Under identical pressure, the charge outputs were 492.7 ± 0.13 pC for CoFe_2_O_4_-PVDF, 131.9 ± 0.36 pC for PVDF, and 77.0 ± 0.81 pC for Fe_3_O_4_-PVDF. The CoFe_2_O_4_-PVDF composite exhibited the highest piezoelectric response, with a charge output approximately four times greater than that of pure PVDF.

Fig. 9d is the differential charge density diagram of CoFe_2_O_4_-PVDF obtained from First-principles simulative calculating. The golden-yellow region represents an increase in electron density after the PVDF molecular chain binds to the CoFe_2_O_4_ crystal, whereas blue regions correspond to a decrease in electron density. As shown in Fig. 9d, the electron cloud density around the atoms of the PVDF molecular chain either increased or decreased, indicating the evident charge exchange among atoms. Fig. 9e and f shows the density of states (DOS) of CoFe_2_O_4_-PVDF and PVDF, respectively. The horizontal axis ranges from −1 eV to 1 eV, with the Fermi level located at 0 eV [36], while the vertical axis represents the atomic density of states. Each curve in the figure represents the density of states of the corresponding atom at the Fermi level. The results of Bader charge analysis are presented in Table 2 of the Supplementary Materials. Bader charge is a calculation method that decomposes a molecule into atoms and then defines the atoms based on the charge density [37,38]. The symbol “+” denotes the acquisition of positive charge, while “-” denotes the acquisition of negative charge. As depicted in Fig. 9f, the density of states of PVDF near the Fermi level was relatively low, but the density of states of CoFe_2_O_4_-PVDF near the Fermi level increased after doping with CoFe_2_O_4_, as presented in Fig. 9e. Fig. 9g and h displays the electron work functions of CoFe_2_O_4_-PVDF and PVDF, respectively. The work function, defined as the minimum energy required to remove an electron from the solid surface, corresponds numerically to the energy difference between the vacuum level and the Fermi level [37]. The work function of CoFe_2_O_4_-PVDF and PVDF were, 2.946 eV and 6.395 eV as shown in Fig. 9g and h, respectively. Therefore, the work function of CoFe_2_O_4_-PVDF was markedly lower than that of PVDF.

## 4 Discussion

In this study, we developed an on-demand power-suppliable system composed of magnetostrictive CoFe_2_O_4_ and PVDF via magneto-responsive-piezoelectricity. After implanted this power-suppliable system into the heart of myocardial infarction model rat, the local electrical micro-environment could be precisely regulated by a non-invasive external magnetic field stimulation to solve the problem of uncontrollable electric signals for traditional piezoelectric materials. Particularly, therapeutic bioelectrical signals would be generated in situ through magnetostrain-piezoelectric conversion as the on-demanded manner. Thus, this power-suppliable system significantly improved cardiac function and reduced fibrosis area *in vivo* via up-regulated angiogenesis, calcium signaling and muscle related genes. It also enhanced the activity and function of injured myocardial cells *in vitro*.

Ischemic heart disease (IHD), especially myocardial infarction (MI) as one of frequent and severe cardiovascular diseases, result from the irreversible injure of myocardial cells induced by apoptotic and necrotic events due to the insufficient blood supply [39]. Injured myocardium forms non-conductive scar tissue, which hinders their electrical conduction and contraction as normal myocardium [40]. Thus, the developed on-demand CoFe_2_O_4_-PVDF power-suppliable system was innovatively implanted into the heart of myocardial infarction model rat via thoracotomy and subsequently fixed with the pericardium in this study. Our experimental results showed that the charge output of CoFe_2_O_4_-PVDF was increased by approximately fourfold compared to that of PVDF (Fig. 9c). Furthermore, the calculation of First-principles revealed that the density of states for CoFe_2_O_4_-PVDF were distinctly increased with the adding of CoFe_2_O_4_ compared with that of PVDF. Additionally, the work function of CoFe_2_O_4_-PVDF and PVDF were 2.946 and 6.395 eV, respectively. The lower the work function means that an electron energy is easy to escape from the surface of a solid. Thus, the present experimental and calculation results indicated the implanted CoFe_2_O_4_-PVDF would generate the extra local charge output to surround injured myocardium under the stimulation of external magnetic field as an on-demanded member. Myocardial cells are electroactive cells, which are governed by electrical signals. Hence, these electronic cues would activate the electroactivity of injured myocardial cells via an intracellular calcium-dependent process, the excitation-contraction coupling [12,23]. Subsequently, the electrical signal stimulation and contraction in the infarction area and injured myocardium might be improved, respectively.

In fact, the myocardium experiences the adverse remodeling at the end stage with the myocardial infarction repairing partial [41]. Specifically, to compensate for the function of scar tissue, normal myocardium industriously enhances the maintenance of the heart contraction and relaxation [12]. Nevertheless, the ventricular wall will become hypertrophic as scar tissue lacks the function of electrical conduction and contraction. Ultimately, it would lead to heart failure [42]. In clinic, the ejection fraction is defined as the percentage of the stroke volume in the end-diastolic volume of the ventricle, which is regularly utilized to assess the heart function of patient. The higher the ejection fraction implies a better pumping function of myocardium [43]. Thus, the ejection fraction was used to assess the influence of the CoFe_2_O_4_-PVDF power-suppliable system on infarcted myocardium. The ejection fraction of myocardial infarction model rat significantly increased by 20.9% after implanted, which dynamically responded to the external magnetic field stimulation for half an hour at each day for 28 days (Fig. 2c). Correspondingly, Fig. 2e confirmed that the fibrotic area of the infarcted myocardium was significantly diminished. The present results demonstrated that the CoFe_2_O_4_-PVDF power-suppliable system under external magnetic field stimulation had a substantial impact on the improvement of myocardium as an on-demanded manner. Additionally, this beneficial effect was attributed to the material stability of CoFe_2_O_4_-PVDF *in vivo*. As shown in Fig. 1g, CoFe_2_O_4_-PVDF still retained a fibrous morphology with the presence of Co and Fe elements after being implanted in the myocardial infarction model rats for 28 days, which was due to the non-degradable PVDF and insoluble CoFe_2_O_4_. Particularly, the β-PVDF phase of the CoFe_2_O_4_-PVDF power-suppliable system remained stable even after being implanted for 28 days *in vivo* as shown in Fig. 1c and d. It demonstrated that the piezoelectric performance of the CoFe_2_O_4_-PVDF power-suppliable system remained stable and can respond to external magnetic field to regenerate the charge putout after being implanted *in vivo*.

Fig. 4d, h, and 4l present the quantitative immunofluorescence intensity data for Piezo1, eNOS, and VEGF in HUVECs exposed to CoFe_2_O_4_-PVDF under external magnetic stimulation. All three demonstrate statistically significant upregulation of these proteins relative to control groups, consistent with the enrichment of angiogenesis and calcium signaling, related terms identified in the GO and KEGG pathway analyses (Fig. 3c and d). Based on these convergent experimental and bioinformatic findings, we propose a mechanistic model for magnetoelectric-mediated cardiac repair: Piezo1, as a primary mechanosensor, detects the coupled piezoelectric and magnetic stimuli generated by CoFe_2_O_4_-PVDF, triggering Ca^2+^ influx through membrane-associated channels; this calcium signal acts as a second messenger to activate downstream effectors, including eNOS phosphorylation and VEGF transcriptional upregulation, thereby driving endothelial proliferation, capillary network formation, and ultimately, paracrine-mediated cardiomyocyte repair. A schematic summary of this proposed cascade is illustrated in Fig. 4n.

Generally, there are "gap junction" channels among cardiomyocytes [44], which direct transmission of electrical signals among cardiomyocytes. As a result, cardiomyocytes can engage in electrical signal cooperation, ultimately leading to synchronous contraction [44,45]. Among them, protein conexin43 (Cx43) serves as a crucial regulatory factor for connecting and mediating the coupling among cardiomyocytes [46,47]. The positive area of Cx43 in the myocardial tissue of model rats was significantly increased by synergistic effect of CoFe_2_O_4_-PVDF energy supply system and external magnetic field as shown by in Fig. 6b and c. In clinical, myocardial infarction is diagnosed by measuring cardiac troponin in tissues or blood [48]. Among these, cardiac troponin T (cTnT) is exclusively expressed in cardiomyocytes and features a distinct cardiac-specific amino acid sequence. It is the preferred biomarker for detecting myocardial infarction or myocardial injury [49]. In Fig. 6d, the brown region represented the cTnT positive markers released from the injured myocardium. Specifically, the more severely the myocardium was injured, the greater the amount of cTnT positive markers released. As depicted in Fig. 6d_5_, the area of cTnT positive markers in the infarcted myocardium was significantly diminished after treatment with the CoFe_2_O_4_-PVDF power-suppliable system and external magnetic field. These results demonstrated that the CoFe_2_O_4_-PVDF power-suppliable system and external magnetic field could improve the infarcted myocardium in rats partially.

**Fig. 10:**
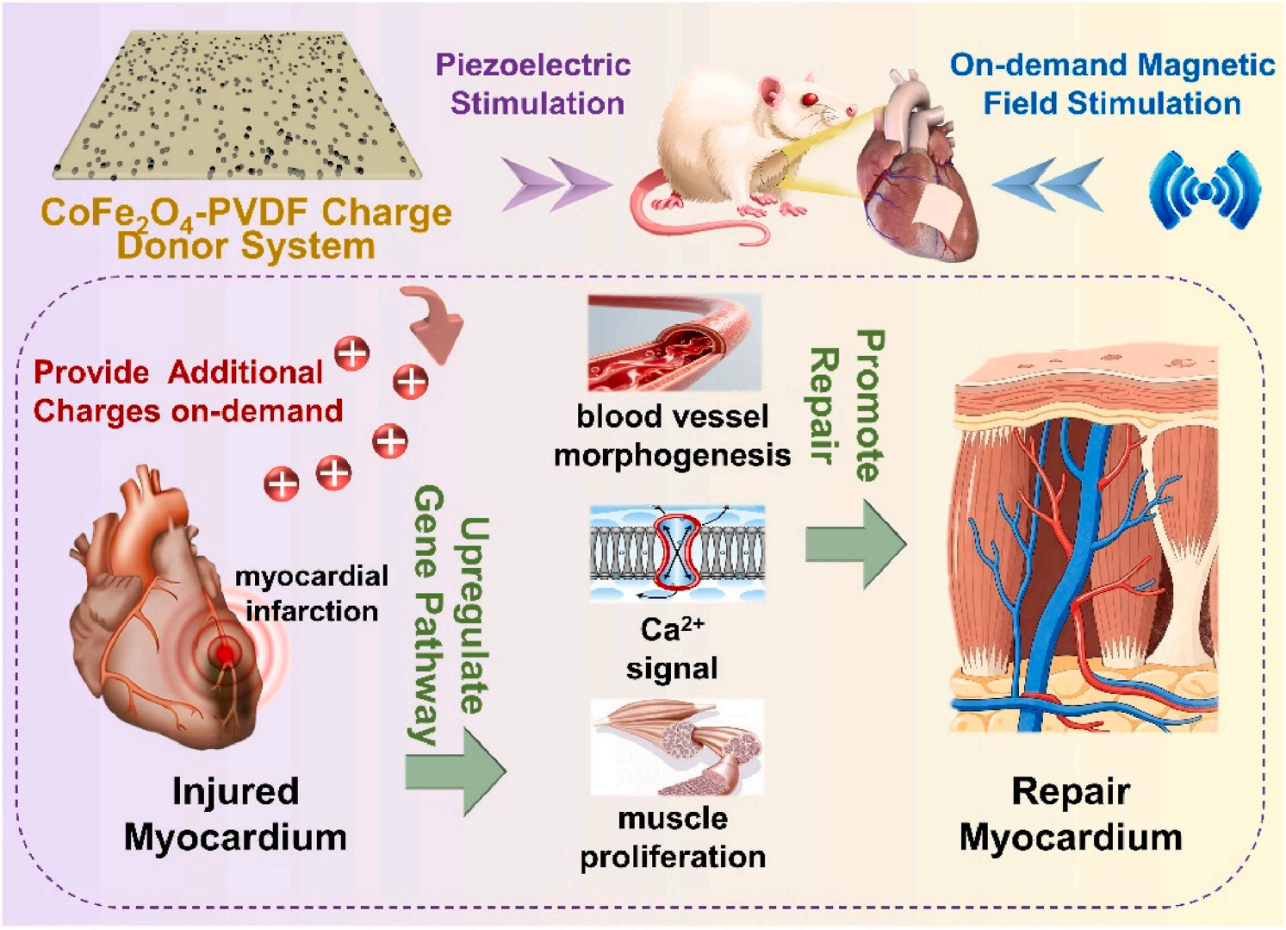
Schematic illustration of the biological mechanism on improving infarcted myocardium treated by the CoFe_2_O_4_-PVDF power-supplying system and the external magnetic field. Postoperatively, rats were subjected to non-invasive external magnetic field stimulation to produce the on-demand modulation of local electrical microenvironment, as the magnetostrictive strain of CoFe_2_O_4_ was precisely converted into piezoelectric charges by PVDF. It up-regulated the genes associated with the “blood vessel morphogenesis”, “angiogenesis”, and “regulation of angiogenesis”, promoted the vascularization of the injured myocardium, and thereby enhanced infarcted myocardium.

Additionally, GO and KEGG analyses of transcriptome from infarcted myocardium in model rats revealed that there were three kinds of up-regulated genes related to angiogenesis promotion, calcium signal and muscle cell proliferation, after implanted and treated with the CoFe_2_O_4_-PVDF power-suppliable system and an external magnetic field for 28 days (Fig. 3c and d). VEGF is a family of factors associated with angiogenesis [50]. Its main functions are to regulate the division and migration of endothelial cells, as well as vasodilation [50]. VEGF typically binds to the receptor protein VEGF-R to exert its biological functions [51]. Western blotting of VEGF-R protein showed that its expression was significantly upregulated following treatment with the CoFe_2_O_4_-PVDF power-suppliable system and external magnetic field compared to that of untreated group as shown in Fig. 3e. This verified the upregulated VEGF gene pathway as the GO biological process analysis. Meanwhile, immunohistochemistry results showed the levels of angiogenesis-related VEGF-A, VEGF-R, and CD31 were significantly upregulated in the Mag + PVDF group as depicted in Fig. 5. It validated the accuracy of the upregulated genes associated with the biological processes of “blood vessel morphogenesis”, “angiogenesis” and “regulation of angiogenesis” in the GO (Fig. 3c), and "VEGF signaling pathway" in the KEGG (Fig. 3d). It was hypothesized that the CoFe_2_O_4_-PVDF power-suppliable system and the external magnetic field would improve infarcted myocardium by promoting angiogenesis and restoring blood supply, as shown in the schematic diagram of Fig. 10.

It is noteworthy that the upregulated genes included the biological process of “steroid hormone synthesis” and “calcium signaling pathways” in the infarcted myocardium with treated with the CoFe_2_O_4_-PVDF power-supplying system and the external magnetic field by GO and KEGG analysis as shown in Fig. 3c and d. This finding was consistent with the transcriptome results of our previous research on the *in vitro* improvement of doxorubicin-induced oxidative stress injured cardiomyocytes using CoFe_2_O_4_-PVDF [23]. The synthesized steroid hormones bind to the sodium-potassium pump on the cell membrane, open Ca^2+^ channels, raising intracellular Ca^2+^ levels, promote cell contraction, and ultimately improve the injured myocardium. Thus, the present *in vivo* transcriptome results are similar to that of our previous *in vitro* study, in which the CoFe_2_O_4_-PVDF power-supplying system and the external magnetic field improved injured cardiomyocytes and tissues both *in vivo* and *in vitro* by actuating Na^+^ K^+^-ATPase.

Muscle cells are typically divided into three main categories: skeletal muscle cells, cardiac muscle cells, and smooth muscle cells, based on their structure and function [52]. Cardiac muscle cells form the walls of the heart and are characterized by their autorhythmicity, operating under involuntary control [53]. Smooth muscle cells make-up the walls of internal organs (such as the digestive tract and blood vessels) and are also under involuntary control [54]. The present bioinformatics results analysis indicates that genes related to muscle cell and smooth muscle cell proliferation, regulation of actin cytoskeleton were significantly upregulated. Considering the properties of the specimen, it is suggested that the genes related to muscle cell proliferation have been upregulated, which originated from the myocardial cells or smooth muscle cells in the vascular wall. It was indicated that the implanted the CoFe_2_O_4_-PVDF power-suppliable system and the external magnetic field had potential to promote the proliferation of myocardial cells or smooth muscle cells.

In addition, hypoxia injured H9c2 cells simulated the hypoxic injured cardiomyocytes after myocardial infarction by the deoxidizer Na_2_S_2_O_4_ [55,56]. Co-cultured with CoFe_2_O_4_-PVDF combined with external magnetic field could significantly promote the spreading and decrease the apoptosis rates of hypoxic injured H9c2 cells as shown in Fig. 7. Benefiting from the design and construction of the CoFe_2_O_4_-PVDF power-supplying system, its synergy with external magnetic field facilitated the improvement of infarcted myocardium of rats and hypoxic injured H9c2 cells. However, it is still clear how CoFe_2_O_4_ enriched the electronic state of PVDF.

The results of the First-principles simulation calculation showed that the electronic state density of PVDF were enhanced with the adding of CoFe_2_O_4_ under the stimulation of the external magnetic field. Each curve depicts the density of states (DOS) of electrons on the corresponding atom in Fig. 9e and f. The DOS represents the number of electrons within a unit energy range [57]. To a certain extent, the DOS (Fig. 9e and f) serves as a quantitative representation method for the differential charge density diagram (Fig. 9d). Nevertheless, the movement of electrons is extremely frequent and intense [58]. Fermi level can describe the movement of electrons [59]. The Fermi level refers to the highest energy level occupied by electrons in the solid energy band at absolute zero temperature [60]. Investigate the density of states in the vicinity of the Fermi level, which can reflect the electron motion state of the material [60]. Specifically, the Fermi level lies in the range of −1 eV to 1 eV on the abscissa in Fig. 9e and f. The DOS of PVDF near the Fermi level was relatively low. Nevertheless, the DOS near the Fermi level increased after doping PVDF with CoFe_2_O_4_ (Fig. 9e) under the external magnetic field. The reason is that the H atom has a small number of electrons, and the F atom exhibits strong electronegativity, leading to a high degree of electron localization in PVDF molecule [58]. Consequently, nearly all the electrons near the Fermi level are contributed by C atoms [58]. When CoFe_2_O_4_ was incorporated, the Co, Fe, and O atoms improved the electronic states of PVDF in the vicinity of the Fermi level and increased the electron density near the Fermi level. This was consistent with the Bader charge calculation results presented in Table 2 of the Supplementary Materials and the calculation results of the differential charge density (Fig. 9d). In addition, the work function is defined as the energy level difference between the vacuum level and the Fermi level [36]. It serves as a measure of the minimum energy necessary for electrons to escape from the surface of a solid. Numerically, the work function is equivalent to the energy difference between the vacuum energy level and the Fermi energy level [36]. The work function decreased from 6.395 eV (PVDF, Fig. 9h) to 2.946 eV (CoFe_2_O_4_-PVDF, Fig. 9g) under the external magnetic field. As is known, the smaller the work function, the less energy is required for electron transition, and thus the easier the electron transition occurs [36]. Therefore, the electrons in CoFe_2_O_4_ migrated to PVDF enriching the electronic state of CoFe_2_O_4_-PVDF under the external magnetic field. The external magnetic field, as a non-intrusive and effective regulation method, not only enhanced the piezoelectric performance of PVDF but also optimized the parameters of external magnetic field stimulation as needed, enabling on-demand supply of electrical energy to injured myocardium.

## 5 Conclusion

In summary, this study successfully developed an on-demand CoFe_2_O_4_-PVDF power-suppliable system for the treatment of infarction myocardium under the adjustable non-invasive external magnetic field, which improved local bioelectrical micro-environment and facilitated the functional recovery of the injured myocardium. The principal findings are summarized as follows:

(1) The on-demand piezoelectricity of CoFe_2_O_4_-PVDF improved the cardiac function and reduced fibrosis area of infracted myocardium *in vivo*

The CoFe_2_O_4_-PVDF system possessed the stable piezoelectricity and structure over 28 days postoperatively. On-demand piezoelectric charges can be generated by applying an external magnetic field. As a result, it significantly improved key cardiac functional parameters, notably the ejection fraction, and simultaneously reduced the fibrotic area in the infarcted myocardium.

(2) The mechanism of improved myocardial tissue is associated with upregulated three kinds of genes, angiogenesis, calcium signal and muscle proliferation

Bioinformatics analysis revealed that CoFe_2_O_4_-PVDF markedly upregulated three kinds of genes associated with angiogenesis, calcium signal and muscle proliferation under external magnetic field *in vivo*. This pro-angiogenic effect was further confirmed by immunohistochemistry and fluorescence staining, which contributed to restoring blood supply to the infracted myocardium.

(3) *In Vitro* Amelioration of Hypoxic-Injured Cardiomyocytes

The CoFe_2_O_4_–PVDF and an external magnetic field could effectively ameliorate hypoxia-induced injured H9c2 cardiomyocytes under external magnetic field actuation. It significantly reduced the apoptosis rate by 5.13%.

(4) Fundamental Enhancement Mechanism

First-principles simulation elucidated that the doped CoFe_2_O_4_ could increase the electronic state density of PVDF and reduced the work function, thereby decreasing the energy barrier for electron output under external magnetic field. The doped CoFe_2_O_4_ could not only enhance the piezoelectricity of PVDF but also increase the charge output. The CoFe_2_O_4_-PVDF power-suppliable system could on-demand generate electrical charges under adjustable external magnetic field actuation, thereby it is potential for improving the disorder electrical micro-environment of the infarcted myocardium.

The present results not only provided a novel on-demand power-suppliable strategy for infarcted myocardial repair but also explored the biological therapeutic mechanisms, paving the way for future bioelectronic therapy.

## CRediT authorship contribution statement

**Tao Jing:** Formal analysis, Investigation, Visualization, Writing – original draft. **Yaolei Zhang:** Investigation, Validation. **Zhongtao Li:** Methodology, Validation. **Xiong Xiong:** Methodology. **Hongyu Sun:** Methodology, Resources. **Yonghong Fan:** Methodology. **Chaoming Xie:** Methodology, Resources. **Feng Lin:** Methodology. **Jie Weng:** Methodology. **Shuxin Qu:** Conceptualization, Supervision, Writing – review & editing.

## Declaration of competing interest

The authors declare that they have no known competing financial interests or personal relationships that could have appeared to influence the work reported in this paper.

## Data Availability

Data will be made available on request.
