# Supplementary material for: Magneto-responsive piezoelectric CoFe2O4-PVDF system for on-demand electrical modulation of infarcted myocardium
Source: Mater Today Bio. 2026 Aug 3;40:103500. doi: 10.1016/j.mtbio.2026.103500 (PMC13473950; doi:10.1016/j.mtbio.2026.103500)
Supplement: Supplementary file 1 [file mmc2.docx]

| Table 1. Surgery and grouping of rats | | | | |  |
| --- | --- | --- | --- | --- | --- |
| **NO.** | **Name** | **Ligation the coronary artery** | **Implant material** | **Magnetic field stimulation** | **Number** |
| 1 | Sham | **×** | **×** | **×** | 6 |
| 2 | MI | √ | **×** | **×** | 6 |
| 3 | Mag | √ | **×** | √ | 6 |
| 4 | PVDF | √ | √ | **×** | 6 |
| 5 | Mag+PVDF | √ | √ | √ | 6 |

| Table 2. Results of Bader charge calculation | | | | | | |
| --- | --- | --- | --- | --- | --- | --- |
| **Material** | **C** | **H** | **F** | **O** | **Fe** | **Co** |
| PVDF | -0.5616 | 0.0083 | 0.5001 | / | / | / |
| CoFe_2_O_4_-PVDF | -0.3529 | 0.4982 | 0.9871 | 1.8318 | -1.2605 | -0.8562 |
